# Sumoylation, Phosphorylation, and Acetylation Fine-Tune the Turnover of Plant Immunity Components Mediated by Ubiquitination

**DOI:** 10.3389/fpls.2017.01682

**Published:** 2017-10-10

**Authors:** Zhouqing He, Tingting Huang, Kevin Ao, Xiaofang Yan, Yan Huang

**Affiliations:** ^1^College of Life Sciences, Sichuan Agricultural University, Ya’an, China; ^2^Michael Smith Laboratories, University of British Columbia, Vancouver, BC, Canada; ^3^Department of Botany, University of British Columbia, Vancouver, BC, Canada

**Keywords:** plant immunity, sumoylation, phosphorylation, acetylation, ubiquitination, ubiquitin, elongating enzyme E4

## Abstract

Ubiquitination-mediated protein degradation plays a crucial role in the turnover of immune proteins through rapid alteration of protein levels. Specifically, the over-accumulation of immune proteins and consequent activation of immune responses in uninfected cells is prevented through degradation. Protein post-translational modifications can influence and affect ubiquitination. There is accumulating evidence that suggests sumoylation, phosphorylation, and acetylation differentially affect the stability of immune-related proteins, so that control over the accumulation or degradation of proteins is fine-tuned. In this paper, we review the function and mechanism of sumoylation, phosphorylation, acetylation, and ubiquitination in plant disease resistance responses, focusing on how ubiquitination reacts with sumoylation, phosphorylation, and acetylation to regulate plant disease resistance signaling pathways. Future research directions are suggested in order to provide ideas for signaling pathway studies, and to advance the implementation of disease resistance proteins in economically important crops.

## Introduction

Plants mainly rely on two conceptual layers of immune systems to prevent infection and disease. The first system, PTI (PAMP-triggered immunity), involves the detection of PAMPs (pathogen-associated molecular patterns) through PRRs (pattern-recognition receptors). The second layer, ETI (effector-triggered immunity), specifically recognizes effector proteins that pathogens secrete to suppress PTI and achieve virulence. ETI involves the detection of effectors through R (resistance) proteins that can induce a rapid and robust immune response (Jones and Dangl, 2006; Boller and He, 2009).

Although plant immune receptors can confer resistance, their over-accumulation or constitutive activity can impair plant growth and development and result in autoimmune phenotypes in the absence of pathogens (van Wersch et al., 2016). For example, *snc1-1 (suppressor of npr1-1, constitutive 1)*, a gain-of-function mutant in an immune receptor-coding gene, exhibits constitutive defense activation without pathogen attack, resulting in autoimmune phenotypes which include dwarf stature, increased SNC1 protein stability, high defense marker gene expression, elevated defense hormone SA (salicylic acid) accumulation and disease resistance (Zhang et al., 2003; Cheng et al., 2011). Therefore, in order to balance appropriate plant immune signaling with normal growth and development, the transient levels of immune receptors in the cell must be tightly controlled. However, the molecular mechanisms that regulate the abundance of receptor proteins are still unclear (Li et al., 2015).

In eukaryotes, PTMs (protein post-translational modifications), such as sumoylation, phosphorylation, acetylation, and ubiquitination, can result in the generation or adjustment of important molecular signals. There are several examples in plant immunity where these modifications have been reported to participate in the regulation of plant disease-resistant immune signals (Cheng et al., 2011; Huang et al., 2014; Eiyama and Okamoto, 2015; Mach, 2015; Saleh et al., 2015; Xu et al., 2015; Zhou et al., 2015; Copeland et al., 2016; Gou et al., 2017). In these modalities, ubiquitination is considered a hub for the regulation of plant immune signals (Cheng and Li, 2012).

The ubiquitination process is an ATP-dependent conjugation cascade involving a series of enzymes, including an ubiquitin activating enzyme E1, an ubiquitin conjugating enzyme E2, and an ubiquitin ligase E3. Usually, an E1 activates Ub (ubiquitin) and transfers it to an E2. An E3 connects a substrate that it specifically recognizes to the Ub molecule conjugated by the E2 (Sadanandom et al., 2012; Metzger et al., 2014). In addition, there is an ubiquitin elongating enzyme E4 that contributes to poly-ubiquitination (Liu et al., 2010; Huang et al., 2014; Ferreira et al., 2015).

In the process of ubiquitination, the immune proteins are specifically recognized by ubiquitin ligase E3s. However, a number of studies have found that the specific recognition of immune proteins is not solely determined by the corresponding ubiquitin ligase E3s, and can require further substrate modifications (Spoel et al., 2009; Lu et al., 2011; Xu et al., 2015). The homeostatic regulation of immune protein is more sophisticated and complex than current models suggest. Upstream of ubiquitination, a variety of protein post-translational modifications, serve as degrons for ubiquitination and ubiquitin-dependent degradation (Yoo et al., 2008; Praefcke et al., 2012; Xu et al., 2015). In this review, we summarize recent advances in the regulation of plant immune protein turnover, and propose an updated model on the control pathways of immune receptors through sumoylation, phosphorylation, acetylation, and ubiquitination.

## Sumoylation

Sumoylation is a PTM that involves the attachment of SUMO (small-ubiquitin-like modifier) proteins to specific lysine residues on substrates. The process by which SUMO chains form and the three-dimensional structure of SUMO proteins is similar to ubiquitination. However, sumoylation seems to be involved in different biological processes compared to ubiquitination, such as genome stability, protein sorting and activation, signal transduction, cell cycle, etc. (Baczyk et al., 2017; Nair et al., 2017; Qiu et al., 2017; Wei et al., 2017). The functional study of SUMO proteins in plants began in 1999 when a novel tomato SUMO protein, with homology to human SUMO1, was found to inhibit EIX-induced (ethylene-inducing xylanase) cell death (Hanania et al., 1999). With the discovery and characterization of two SUMO E3 coding genes *SIZI* (*SAP* and *MIZ1*) and *MMS21*/*HPY2* (*Methyl Methanesulfonate-sensitivity Protein 21*/*High Ploidy 2*) in *Arabidopsis thaliana*, as well two SUMO E4 coding genes *PIAL1* and *PIAL2* (*Protein Inhibitor of Activated STAT Like1/2*), the roles of sumoylation in regulating plant processes are emerging (Miura et al., 2005; Huang et al., 2009; Ishida et al., 2012; Tomanov et al., 2014; Kwak et al., 2016). In plant immunity, SUMO modification seems to play both positive and negative regulatory roles (Lee et al., 2007; van den Burg and Takken, 2010; Bailey et al., 2016; Cheng et al., 2017).

Current models suggest that there are three main relationships between sumoylation and ubiquitination: antagonism, synergism or feedback, and degron mediation. Firstly, sumoylation and ubiquitination can antagonistically compete for the same lysine residue of target substrates (Hoege et al., 2002; Ulrich, 2005). In some cases antagonistic effects can also be observed between sumoylation and ubiquitination when the modification site is not the same (Desterro et al., 1998; Gostissa et al., 1999). Secondly, ubiquitination and sumoylation can affect one another positively in a feedback loop. For example, SUMO E3 SIZ1 can enhance the transubiquitination activity of COP1 (constitutive photomorphogenic 1), an ubiquitin E3 ligase, but its own protein level and stability is modulated by COP1 (Lin et al., 2016). Thirdly, sumoylation can function as a degron mediating ubiquitin-dependent degradation by the proteasome (Miteva et al., 2010; Manente et al., 2011; Praefcke et al., 2012). STUbLs (SUMO-targeted ubiquitin ligases), identified in yeast, humans and plants, regulate the level and stability of sumoylated proteins and act downstream of sumoylation (Xie et al., 2007; Tatham et al., 2008; Elrouby et al., 2013).

In plant immunity, there are two immunity-related studies supporting that sumoylated residues act as degrons for ubiquitous degradation in plants. Firstly, SIZ1 was found to contribute to the turnover of SNC1 and play a role in plant immunity. The loss-of-function mutant of *SIZ1* exhibited autoimmune phenotypes that are partly dependent on SNC1, and over-expression of CPR1 (constitutive expressor of PR genes 1), an E3 ubiquitin ligase previously reported to target SNC1 for degradation, could restore the growth defects caused by *siz1* (Gou et al., 2012, 2017). SIMs (SUMO-interaction motifs) are sequences that can mediate the binding of proteins to sumoylated substrates. For example, sumoylated PCNA (proliferating-cell nuclear antigen) can be recognized by the SIM of the ubiquitin ligase Rad18 (radiation-sensitive 18) in yeast (Parker and Ulrich, 2014). In SNC1, there are four sumoylation sites and five putative SIMs (Gou et al., 2017). It would be interesting to determine whether sumoylation enhances SNC1 turnover, increasing its ubiquitination and proteasome-mediated degradation.

A second example involves the key SA receptor and transcriptional activator, NPR1 (non-expressor of PR genes 1). Under normal conditions, NPR1 mainly exists in the cytoplasm as an oligomer, but a small amount of NPR1 monomer can be sumoylated, transported to the nucleus, and degraded through ubiquitination-mediate pathways. This process maintains NPR1-mediated basic immunity and restricts its co-activator activity to prevent autoimmunity. In the presence of pathogens, accumulation of SA triggers the reduction of NPR1 oligomers to monomers, the sumoylation of the monomers, and finally the translocation of the monomers into the nucleus to promote defense gene transcription. NPR1 contains a BTB/POZ (for Broad Complex, Tramtrack and Bric-a-brac/Pox Virus and Zinc Finger) domain, which functions as a substrate adapter of SCF (Skp1-cullin1-F-box) -type ubiquitin ligase E3s (Rochon et al., 2006). Interestingly, BTB-type proteins themselves may also be substrates for SCF complexes (Boyle et al., 2009). NPR1 can be phosphorylated at residues Ser11/Ser15 to facilitate further sumoylation of NPR1 and promote its recruitment to Cullin-based ubiquitin ligase E3 for degradation (Spoel et al., 2009; Mach, 2015; Saleh et al., 2015). It is likely that the ubiquitination of mission-capable NPR1 from the target gene promoter promotes NPR1 in the cytoplasm to translocate to the nucleus to launch a fresh transcription cycle.

## Phosphorylation

Phosphorylation refers to the addition of a phosphoric acid group to proteins or other functional molecules. Protein phosphorylation is a reversible reaction that occurs mainly on serine, threonine, and tyrosine residues. The main role of serine and threonine phosphorylation is to alter the protein activity state from inactive to active (Rahimi and Costello, 2015). Tyrosine phosphorylation, on the other hand, generally provides a structural group to facilitate interactions with other proteins, in order to form poly-protein complexes that further promote protein phosphorylation in a phosphorylation cascade (Kobayashi et al., 2014). The signal generated is eventually transferred to the nucleus, leading to transcriptional changes (Gao and He, 2013). There is emerging evidence that phosphorylation plays a role upstream of ubiquitination in plant innate immunity (Lu et al., 2011; Saleh et al., 2015; Swaney et al., 2015).

*Arabidopsis* BIK1 (botrytis-induced kinase 1) is a cytosolic kinase that phosphorylates and enhances the activity of BAK1 (BRI1-associated receptor kinase 1). A yeast two-hybrid screen done using BAK1 as bait isolated PUB13 (plant U-box 13), an ubiquitin ligase E3, which possesses an N-terminal U-box domain. Further studies show that BAK1 phosphorylates PUB13 and its close homolog PUB12, and that this phosphorylation is required in order for the E3s to associate with and ubiquitinate FLS2 (flagellin sensitive 2), a plasma-membrane-localized PRR protein (Gomez-Gomez and Boller, 2000; Lu et al., 2011; Zhou et al., 2015). In this example, the FLS2-mediated immune response is regulated by a phosphorylation cascade pathway, which determines which proteins to degrade via ubiquitination and which to maintain for sustained immune signaling.

Rice PRR protein Xa21 (*Xanthomonas oryzae* pv. Oryzae 21) has many auto-phosphorylated sites in the JM (juxtamembrane) domain, and can interact with a RING-type ubiquitin ligase XB3 (Xa21 binding protein 3), which maintains the stability of Xa21 (Wang et al., 2006; Xu et al., 2006). Recent studies found that Thr-705 in the JM domain of Xa21 is essential for its auto-phosphorylation and that mutation in Thr-705 abolished the interaction between Xa21 with XB3, suggesting that Thr-705 plays an important biological function in Xa21-mediated defense. Upon pathogen infection, Xa21 recognizes PAMPs and transfers phosphate groups to XB3, leading to XB3 self-ubiquitination and activation of MAPK (mitogen-activated protein kinase) cascades-mediated resistance (Chen et al., 2010; Park et al., 2010; Huang et al., 2013).

Furthermore, there are more sophisticated phosphorylation mechanisms to regulate defense related proteins in multiple layers. EIN3 (ethylene-insensitive 3), is a plant-specific nuclear transcription factor that functions in MAPK cascades to initiate downstream transcriptional cascades for ethylene responses. EIN3 interacts with two F-box-type ubiquitin ligases, EBF1 (early B-cell factor 1) and EBF2 and is ubiquitinated and degraded by the 26S proteasome. There is a dual phosphorylation modulatory mechanism for EIN3 stability: MKK9 (MAP kinase kinase 9) phosphorylates T174 to promote EIN3 stability whereas CTR1 (copper transporter 1) phosphorylates T592 to facilitate EIN3 degradation (Binder et al., 2007; Yoo et al., 2008; Tacken et al., 2012). Similarly, the phosphorylation sites at Ser11/Ser15 of NPR1 promote its ubiquitin-mediated degradation; while the sites at Ser55/Ser59 maintain NPR1 stability and inactivity (Spoel et al., 2009; Saleh et al., 2015). It would be interesting to investigate whether the phosphorylation sites of Xa21 also have opposing functions.

## Acetylation

It has been well-established that histone acetylation plays a key role in DNA transcription, and that acetylation of non-histones plays an important role in cellular events (Spange et al., 2009; Graff and Tsai, 2013). Recently, more studies have revealed that N-terminal (Nt) acetylation can serve as signals for ubiquitin-mediated protein degradation (Hwang et al., 2010; Gibbs, 2015; Xu et al., 2015). Pathogen effectors can also acetylate immune receptors to promote infection (Song and Walley, 2016).

Akin to the dual roles of phosphorylation in PTI, Nt-acetylation serves two functions in SNC1-mediated immunity (Xu et al., 2015). The Nt-acetylation of SNC1 was first identified in *muse6* (*mutant, snc1-enhancing 6*), a mutant derived from *mos4* (*modifier of snc1 4*) *snc1* background with strong autoimmunity phenotypes. Sequence homology analysis revealed that *Arabidopsis* MUSE6 is an ortholog of Yeast Naa15 [N (alpha)-acetyltransferase 15], the subsidiary subunit of NatA (N-terminal acetyltransferase complex A), suggesting that Nt-acetylation contributes to SNC1 degradation. Interestingly, SNC1 seemed to have distinct N-terminal isoforms as detected by MS analysis, which may have been generated through alternative initiation and Nt-acetylation. Affinity purification and *in vitro* enzymatic assays proved that its first Met is acetylated by NatA serving as a degron for ubiquitination, while the second Met is acetylated by NatB, stabilizing SNC1. The turnover of RPM1 was also linked with NatA, but not NatB (Van Damme et al., 2012; Eiyama and Okamoto, 2015; Xu et al., 2015). Interestingly, the antagonistic function of NatA and NatB is also observed in flowering time (Kapos et al., 2015).

Many N-terminally acetylated amino acids which are targeted by NatA act as degrons in yeast and are recognized by the ubiquitin E3 Doa10 (degradation of Alpha2 10) for degradation (Hwang et al., 2010; Zattas et al., 2013). Thus, it is hypothesized that a single immune protein can be Nt-acetylated in different ways by different Nats, and the acetylation can be differentially recognized by different ubiquitin ligase E3s, providing regulation through two antagonistic downstream routes. However, it has not been confirmed whether acetylation changes the charge state or the spatial structure of SNC1 to expose its ubiquitin recognition site to be recognized by E3; or whether the ubiquitin ligase E3 directly recognizes the acetylated site. Therefore, unlike the diverse mechanisms of interaction between sumoylation and ubiquitination, the relationship between acetylation and ubiquitination or sumoylation remains to be explored in detail. Structural studies of the E3 recognition site will contribute a better understanding to the synergistic mechanisms of acetylation, ubiquitination, and sumoylation.

## Ubiquitination

Ubiquitination plays a significant role in regulating plant immunity. For example, ubiquitin ligase E3s prevent overproduction of immune proteins (Cheng and Li, 2012; Duplan and Rivas, 2014). One example is *Arabidopsis* ubiquitin ligase CPR1, an F-box protein that regulates the resistance pathways mediated by R proteins such as SNC1 and RPS2. The autoimmune phenotypes of *cpr1* are SNC1-dependent, and CPR1 can decrease SNC1 level via ubiquitin-mediated degradation (Cheng et al., 2011; Gou and Hua, 2012).

Since poly-ubiquitination is the most suitable form of ubiquitination for protein degradation, and since the integrated activities of E1s, E2s, and E3s are rarely effective in adding more than three ubiquitin molecules to substrates, there must be a ubiquitin extension factor involved in the poly-ubiquitination of proteins which is named E4 (Ohki et al., 2009; Ordureau et al., 2014). Yeast Ufd2 (ubiquitin fusion degradation protein 2), the first discovered and best characterized E4 factor, has a C-terminal U-box domain, which promotes the assembly of poly-ubiquitin chain cooperating with E1s, E2s, and E3s (Hanzelmann et al., 2010). Yeast Cdc48 (cell division cycle 48), a conserved ATPase among eukaryotes, interacts with Ufd2 and may play an important role in transporting ubiquitous targets to the proteasome (Baek et al., 2011).

At present, many Ufd2 orthologs have been functionally studied: the mouse E4B (also known as Ufd2a), the human GP78 (glycoprotein of Mr 7800) and MUSE3 from *Arabidopsis* (Susaki et al., 2010; Huang et al., 2014; Wang et al., 2014). MUSE3, the only E4 factor in *Arabidopsis* identified from a forward genetic screen in the background of *mos4 snc1*, affects SNC1 and RPS2 protein levels and relies on CPR1 through the UPS (ubiquitin proteasome system) pathway. Co-IP experiments revealed that MUSE3 appears to directly interact with SNC1, but not with RPS2, indicating that the association between MUSE3 and RPS2 may need an unknown intermediary agent (Huang et al., 2014). There is also an ATPase MUSE8 that interacts with MUSE3 to negatively regulate plant immunity (Copeland et al., 2016).

At present, it is hypothesized that E4s functions in E3-E4 or E4-substrate complexes to assist the transfer of ubiquitin molecules from E2s to the targets, or to add ubiquitin molecules to the targets after E3s’ function (Huang et al., 2014; Ferreira et al., 2015). There are more than 1000 genes coding ubiquitin ligase E3s in the *Arabidopsis* genome, with the biological function of most genes uncharacterized. Therefore, further investigation will be necessary to identify other E3s, E4s and their targets, thus to understand the homeostatic regulatory mechanism of immune proteins.

## Conclusion and Perspectives

Over the past 10 years, ubiquitination has emerged to be a vital regulatory mechanism in plant biology that is controlled at multiple levels, even in a single signal response pathway. Moreover, ubiquitin molecules can complete the correct assembly and folding of some proteins through the combination of E1s, E2s, and E3s and participate in the regulation of some protein activity. Therefore, the destruction, modification, or recombination of components in UPS can directly or indirectly affect plant hormonal signaling pathways, gene transcription, morphogenesis, resistance to harsh environments, and competition between plants and pathogens. Although recent research has provided us with a basic understanding of the function of UPS pathways in plant immunity, the biochemical mechanisms and physiological functions of ubiquitination are far from being fully understood.

**FIGURE 1:**
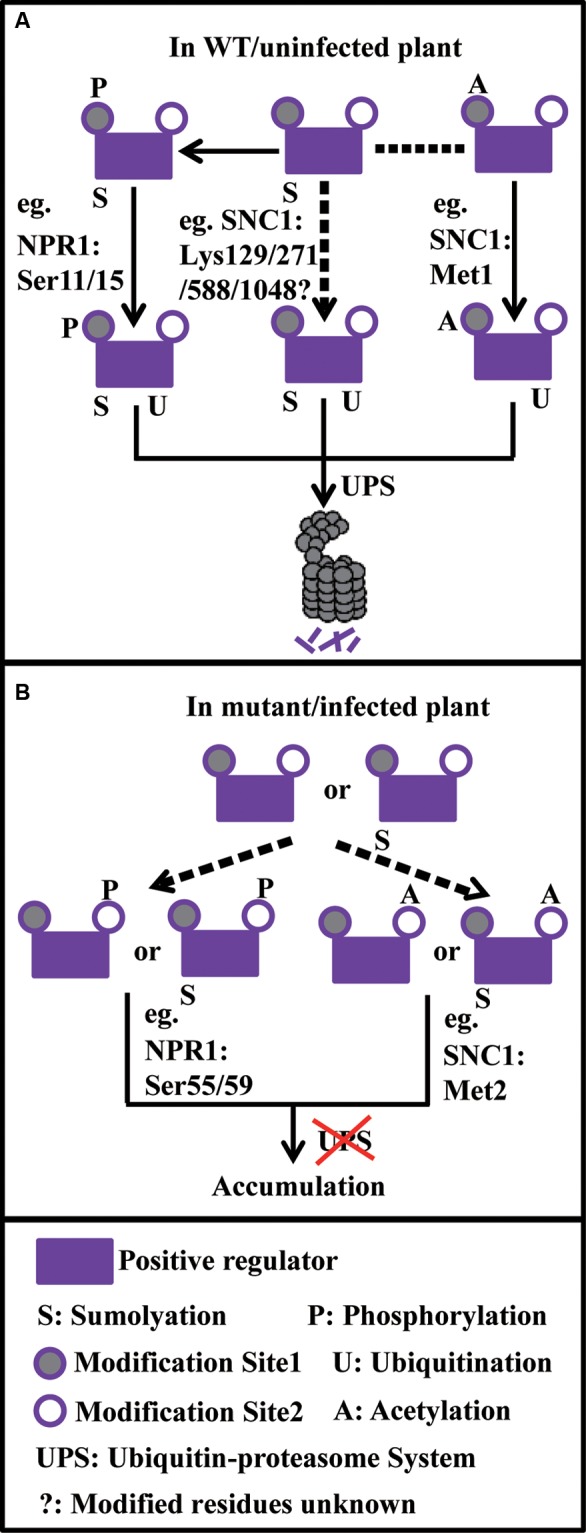
Model on how ubiquitination reacts with sumoylation, phosphorylation, and acetylation to fine-tune the turnover of plant immune components. **(A)** Under normal conditions, sumoylated positive regulators further go through phosphorylation or acetylation on the site1 (e.g., NPR1: Ser11/15 and SNC1: Met1, respectively), which serves as degron, leading to protein degradation through the UPS. Or protein sumoylation alone (e.g., SNC1: Lys129/271/588/1048) may promote its degradation through the UPS. **(B)** Under abnormal conditions, positive regulators or sumoylated positive regulators go through phosphorylation or acetylation on the site2 (e.g., NPR1: Ser55/59 and SNC1: Met2, respectively), which prevents protein degradation through the UPS, leading to protein accumulation. In the model, dash arrows stand for uncertain events.

The existence of a variety of PTMs that work together with ubiquitination is thought to provide flexibility for regulation of target protein involved in plant immunity under dynamic cellular conditions (**Figure 1**). Similar mechanisms may be particularly important during growth and development, stress responses, flowering, etc. to ensure an appropriate concentration of regulatory proteins is reached in a timely manner.

There are still many problems that remain unsolved. One of the biggest challenges is to identify the targets of the E3s that participate in plant immunity. Furthermore, how do E3s identify their substrates? What are the characteristics of these substrates? How is the activity of ubiquitous enzymes itself regulated? What is the relationship between different E3s and E4s? Finally, how are ubiquitination and other PTMs such as sumoylation, phosphorylation, and acetylation related? The questions above remained to be resolved through in-depth cooperative and multi-disciplinary studies on plant immunity. Approaches, such as targeted reverse genetics as well as carefully designed novel forward genetic screens and global protein stability profiling, will enable us to reveal these mysteries.

Future research in this area can uncover the intricate interactions among different PTMs, but can also increase the understanding of the function of the UPS. Moreover, exploring the mechanism of plant defense against pathogen infection through the immune system could lead to ideas that eventually become varities of disease resistance that could be implemented in economically important crops.

## Author Contributions

ZH and TH wrote the draft together and contribute equally to this work. KA took charge of English editing and XY draw the model. YH conceived the review and supervised the whole work. All authors contributed to the revision of the manuscript.

## Conflict of Interest Statement

The authors declare that the research was conducted in the absence of any commercial or financial relationships that could be construed as a potential conflict of interest.

## References

[B1] Baczyk D. Audette M. C. Drewlo S. Levytska K. Kingdom J. C. (2017). SUMO-4: a novel functional candidate in the human placental protein SUMOylation machinery. *PLOS ONE* 12:e0178056. 10.1371/journal.pone.0178056 28545138 PMC5435238

[B2] Baek G. H. Kim I. Rao H. (2011). The Cdc48 ATPase modulates the interaction between two proteolytic factors Ufd2 and Rad23. *Proc. Natl. Acad. Sci. U.S.A.* 108 13558–13563. 10.1073/pnas.1104051108 21807993 PMC3158229

[B3] Bailey M. Srivastava A. Conti L. Nelis S. Zhang C. Florance H. (2016). Stability of small ubiquitin-like modifier (SUMO) proteases OVERLY TOLERANT TO SALT1 and -2 modulates salicylic acid signalling and SUMO1/2 conjugation in *Arabidopsis thaliana*. *J. Exp. Bot.* 67 353–363. 10.1093/jxb/erv468 26494731 PMC4682439

[B4] Binder B. M. Walker J. M. Gagne J. M. Emborg T. J. Hemmann G. Bleecker A. B. (2007). The Arabidopsis EIN3 binding F-Box proteins EBF1 and EBF2 have distinct but overlapping roles in ethylene signaling. *Plant Cell* 19 509–523. 10.1105/tpc.106.048140 17307926 PMC1867343

[B5] Boller T. He S. Y. (2009). Innate immunity in plants: an arms race between pattern recognition receptors in plants and effectors in microbial pathogens. *Science* 324 742–744. 10.1126/science.1171647 19423812 PMC2729760

[B6] Boyle P. Le Su E. Rochon A. Shearer H. L. Murmu J. Chu J. Y. (2009). The BTB/POZ domain of the Arabidopsis disease resistance protein NPR1 interacts with the repression domain of TGA2 to negate its function. *Plant Cell* 21 3700–3713. 10.1105/tpc.109.069971 19915088 PMC2798319

[B7] Chen X. Chern M. Canlas P. E. Jiang C. Ruan D. Cao P. (2010). A conserved threonine residue in the juxtamembrane domain of the XA21 pattern recognition receptor is critical for kinase autophosphorylation and XA21-mediated immunity. *J. Biol. Chem.* 285 10454–10463. 10.1074/jbc.M109.093427 20118235 PMC2856252

[B8] Cheng X. Xiong R. Li Y. Li F. Zhou X. Wang A. (2017). Sumoylation of *Turnip mosaic virus* RNA polymerase promotes viral infection by counteracting the host NPR1-mediated immune response. *Plant Cell* 29 508–525. 10.1105/tpc.16.00774 28223439 PMC5385955

[B9] Cheng Y. T. Li X. (2012). Ubiquitination in NB-LRR-mediated immunity. *Curr. Opin. Plant Biol.* 15 392–399. 10.1016/j.pbi.2012.03.014 22503756

[B10] Cheng Y. T. Li Y. Huang S. Huang Y. Dong X. Zhang Y. (2011). Stability of plant immune-receptor resistance proteins is controlled by SKP1-CULLIN1-F-box (SCF)-mediated protein degradation. *Proc. Natl. Acad. Sci. U.S.A.* 108 14694–14699. 10.1073/pnas.1105685108 21873230 PMC3167521

[B11] Copeland C. Ao K. Huang Y. Tong M. Li X. (2016). The evolutionarily conserved E3 ubiquitin ligase AtCHIP contributes to plant immunity. *Front. Plant Sci.* 7:309. 10.3389/fpls.2016.00309 27014328 PMC4791365

[B12] Desterro J. M. Rodriguez M. S. Hay R. T. (1998). SUMO-1 modification of IkappaBalpha inhibits NF-kappaB activation. *Mol. Cell.* 2 233–239. 9734360 10.1016/s1097-2765(00)80133-1

[B13] Duplan V. Rivas S. (2014). E3 ubiquitin-ligases and their target proteins during the regulation of plant innate immunity. *Front. Plant Sci.* 5:42. 10.3389/fpls.2014.00042 24592270 PMC3923142

[B14] Eiyama A. Okamoto K. (2015). Protein N-terminal acetylation by the NatA complex is critical for selective mitochondrial degradation. *J. Biol. Chem.* 290 25034–25044. 10.1074/jbc.M115.677468 26296886 PMC4599008

[B15] Elrouby N. Bonequi M. V. Porri A. Coupland G. (2013). Identification of Arabidopsis SUMO-interacting proteins that regulate chromatin activity and developmental transitions. *Proc. Natl. Acad. Sci. U.S.A.* 110 19956–19961. 10.1073/pnas.1319985110 24255109 PMC3856783

[B16] Ferreira R. T. Menezes R. A. Rodrigues-Pousada C. (2015). E4-Ubiquitin ligase Ufd2 stabilizes Yap8 and modulates arsenic stress responses independent of the U-box motif. *Biol. Open* 4 1122–1131. 10.1242/bio.010405 26276098 PMC4582114

[B17] Gao X. He P. (2013). Nuclear dynamics of Arabidopsis calcium-dependent protein kinases in effector-triggered immunity. *Plant Signal. Behav.* 8:e23868. 10.4161/psb.23868 23425856 PMC3956488

[B18] Gibbs D. J. (2015). Emerging functions for N-terminal protein acetylation in plants. *Trends Plant Sci.* 20 599–601. 10.1016/j.tplants.2015.08.008 26319188 PMC4601045

[B19] Gomez-Gomez L. Boller T. (2000). FLS2: an LRR receptor-like kinase involved in the perception of the bacterial elicitor flagellin in Arabidopsis. *Mol. Cell* 5 1003–1011. 10911994 10.1016/s1097-2765(00)80265-8

[B20] Gostissa M. Hengstermann A. Fogal V. Sandy P. Schwarz S. E. Scheffner M. (1999). Activation of p53 by conjugation to the ubiquitin-like protein SUMO-1. *EMBO J.* 18 6462–6471. 10.1093/emboj/18.22.6462 10562558 PMC1171709

[B21] Gou M. Hua J. (2012). Complex regulation of an R gene SNC1 revealed by auto-immune mutants. *Plant Signal. Behav.* 7 213–216. 10.4161/psb.18884 22415045 PMC3405709

[B22] Gou M. Huang Q. Qian W. Zhang Z. Jia Z. Hua J. (2017). Sumoylation E3 ligase SIZ1 modulates plant immunity partly through the immune receptor gene SNC1 in Arabidopsis. *Mol. Plant Microbe Interact.* 30 334–342. 10.1094/mpmi-02-17-0041-r 28409535

[B23] Gou M. Shi Z. Zhu Y. Bao Z. Wang G. Hua J. (2012). The F-box protein CPR1/CPR30 negatively regulates R protein SNC1 accumulation. *Plant J.* 69 411–420. 10.1111/j.1365-313X.2011.04799.x 21967323

[B24] Graff J. Tsai L. H. (2013). Histone acetylation: molecular mnemonics on the chromatin. *Nat. Rev. Neurosci.* 14 97–111. 10.1038/nrn3427 23324667

[B25] Hanania U. Furman-Matarasso N. Ron M. Avni A. (1999). Isolation of a novel SUMO protein from tomato that suppresses EIX-induced cell death. *Plant J.* 19 533–541. 10504575 10.1046/j.1365-313x.1999.00547.x

[B26] Hanzelmann P. Stingele J. Hofmann K. Schindelin H. Raasi S. (2010). The yeast E4 ubiquitin ligase Ufd2 interacts with the ubiquitin-like domains of Rad23 and Dsk2 via a novel and distinct ubiquitin-like binding domain. *J. Biol. Chem.* 285 20390–20398. 10.1074/jbc.M110.112532 20427284 PMC2888450

[B27] Hoege C. Pfander B. Moldovan G. L. Pyrowolakis G. Jentsch S. (2002). RAD6-dependent DNA repair is linked to modification of PCNA by ubiquitin and SUMO. *Nature* 419 135–141. 10.1038/nature00991 12226657

[B28] Huang L. Yang S. Zhang S. Liu M. Lai J. Qi Y. (2009). The Arabidopsis SUMO E3 ligase AtMMS21, a homologue of NSE2/MMS21, regulates cell proliferation in the root. *Plant J.* 60 666–678. 10.1111/j.1365-313X.2009.03992.x 19682286

[B29] Huang X. Liu X. Chen X. Snyder A. Song W. Y. (2013). Members of the XB3 family from diverse plant species induce programmed cell death in *Nicotiana benthamiana*. *PLOS ONE* 8:e63868. 10.1371/journal.pone.0063868 23717500 PMC3661601

[B30] Huang Y. Minaker S. Roth C. Huang S. Hieter P. Lipka V. (2014). An E4 ligase facilitates polyubiquitination of plant immune receptor resistance proteins in Arabidopsis. *Plant Cell* 26 485–496. 10.1105/tpc.113.119057 24449689 PMC3963591

[B31] Hwang C. S. Shemorry A. Varshavsky A. (2010). N-terminal acetylation of cellular proteins creates specific degradation signals. *Science* 327 973–977. 10.1126/science.1183147 20110468 PMC4259118

[B32] Ishida T. Yoshimura M. Miura K. Sugimoto K. (2012). MMS21/HPY2 and SIZ1, two Arabidopsis SUMO E3 ligases, have distinct functions in development. *PLOS ONE* 7:e46897. 10.1371/journal.pone.0046897 23056518 PMC3466189

[B33] Jones J. D. Dangl J. L. (2006). The plant immune system. *Nature* 444 323–329. 10.1038/nature05286 17108957

[B34] Kapos P. Xu F. Meinnel T. Giglione C. Li X. (2015). N-terminal modifications contribute to flowering time and immune response regulations. *Plant Signal. Behav.* 10:e1073874. 10.1080/15592324.2015.1073874 26361095 PMC4883885

[B35] Kobayashi H. Saito T. Sato K. Furusawa K. Hosokawa T. Tsutsumi K. (2014). Phosphorylation of cyclin-dependent kinase 5 (Cdk5) at Tyr-15 is inhibited by Cdk5 activators and does not contribute to the activation of Cdk5. *J. Biol. Chem.* 289 19627–19636. 10.1074/jbc.M113.501148 24872417 PMC4094073

[B36] Kwak J. S. Son G. H. Kim S. I. Song J. T. Seo H. S. (2016). Arabidopsis HIGH PLOIDY2 sumoylates and stabilizes Flowering Locus C through its E3 ligase activity. *Front. Plant Sci.* 7:530. 10.3389/fpls.2016.00530 27148346 PMC4837325

[B37] Lee J. Nam J. Park H. C. Na G. Miura K. Jin J. B. (2007). Salicylic acid-mediated innate immunity in Arabidopsis is regulated by SIZ1 SUMO E3 ligase. *Plant J.* 49 79–90. 10.1111/j.1365-313X.2006.02947.x 17163880

[B38] Li X. Kapos P. Zhang Y. (2015). NLRs in plants. *Curr. Opin. Immunol.* 32 114–121. 10.1016/j.coi.2015.01.014 25667191

[B39] Lin X.-L. Niu D. Hu Z.-L. Kim D. H. Jin Y. H. Cai B. (2016). An Arabidopsis SUMO E3 ligase, SIZ1, negatively regulates photomorphogenesis by promoting COP1 activity. *PLOS Genetics* 12:e1006016. 10.1371/journal.pgen.1006016 27128446 PMC4851335

[B40] Liu C. van Dyk D. Xu P. Choe V. Pan H. Peng J. (2010). Ubiquitin chain elongation enzyme Ufd2 regulates a subset of Doa10 substrates. *J. Biol. Chem.* 285 10265–10272. 10.1074/jbc.M110.110551 20159987 PMC2856231

[B41] Lu D. Lin W. Gao X. Wu S. Cheng C. Avila J. (2011). Direct ubiquitination of pattern recognition receptor FLS2 attenuates plant innate immunity. *Science* 332 1439–1442. 10.1126/science.1204903 21680842 PMC3243913

[B42] Mach J. (2015). Phosphorylation and nuclear localization of NPR1 in systemic acquired resistance. *Plant Cell* 27 3291. 10.1105/tpc.15.01020 26672072 PMC4707459

[B43] Manente A. G. Pinton G. Tavian D. Lopez-Rodas G. Brunelli E. Moro L. (2011). Coordinated sumoylation and ubiquitination modulate EGF induced EGR1 expression and stability. *PLOS ONE* 6:e25676. 10.1371/journal.pone.0025676 21998680 PMC3187784

[B44] Metzger M. B. Pruneda J. N. Klevit R. E. Weissman A. M. (2014). RING-type E3 ligases: master manipulators of E2 ubiquitin-conjugating enzymes and ubiquitination. *Biochim. Biophys. Acta* 1843 47–60. 10.1016/j.bbamcr.2013.05.026 23747565 PMC4109693

[B45] Miteva M. Keusekotten K. Hofmann K. Praefcke G. J. Dohmen R. J. (2010). Sumoylation as a signal for polyubiquitylation and proteasomal degradation. *Subcell Biochem.* 54 195–214. 10.1007/978-1-4419-6676-6-16 21222284

[B46] Miura K. Rus A. Sharkhuu A. Yokoi S. Karthikeyan A. S. Raghothama K. G. (2005). The Arabidopsis SUMO E3 ligase SIZ1 controls phosphate deficiency responses. *Proc. Natl. Acad. Sci. U.S.A.* 102 7760–7765. 10.1073/pnas.0500778102 15894620 PMC1140425

[B47] Nair R. R. Patil S. Tiron A. Kanhema T. Panja D. Schiro L. (2017). Dynamic arc SUMOylation and selective interaction with F-Actin-Binding protein Drebrin A in LTP consolidation in vivo. *Front. Synaptic Neurosci.* 9:8. 10.3389/fnsyn.2017.00008 28553222 PMC5426369

[B48] Ohki Y. Funatsu N. Konishi N. Chiba T. (2009). The mechanism of poly-NEDD8 chain formation in vitro. *Biochem. Biophys. Res. Commun.* 381 443–447. 10.1016/j.bbrc.2009.02.090 19245792

[B49] Ordureau A. Sarraf S. A. Duda D. M. Heo J. M. Jedrychowski M. P. Sviderskiy V. O. (2014). Quantitative proteomics reveal a feedforward mechanism for mitochondrial PARKIN translocation and ubiquitin chain synthesis. *Mol. Cell* 56 360–375. 10.1016/j.molcel.2014.09.007 25284222 PMC4254048

[B50] Park C. J. Han S. W. Chen X. Ronald P. C. (2010). Elucidation of XA21-mediated innate immunity. *Cell Microbiol.* 12 1017–1025. 10.1111/j.1462-5822.2010.01489.x 20590657 PMC2906629

[B51] Parker J. L. Ulrich H. D. (2014). SIM-dependent enhancement of substrate-specific SUMOylation by a ubiquitin ligase in vitro. *Biochem. J.* 457 435–440. 10.1042/bj20131381 24224485

[B52] Praefcke G. J. Hofmann K. Dohmen R. J. (2012). SUMO playing tag with ubiquitin. *Trends Biochem. Sci.* 37 23–31. 10.1016/j.tibs.2011.09.002 22018829

[B53] Qiu C. Wang Y. Zhao H. Qin L. Shi Y. Zhu X. (2017). The critical role of SENP1-mediated GATA2 deSUMOylation in promoting endothelial activation in graft arteriosclerosis. *Nat. Commun.* 8:15426. 10.1038/ncomms15426 28569748 PMC5461500

[B54] Rahimi N. Costello C. E. (2015). Emerging roles of post-translational modifications in signal transduction and angiogenesis. *Proteomics* 15 300–309. 10.1002/pmic.201400183 25161153 PMC4297243

[B55] Rochon A. Boyle P. Wignes T. Fobert P. R. Despres C. (2006). The coactivator function of Arabidopsis NPR1 requires the core of its BTB/POZ domain and the oxidation of C-terminal cysteines. *Plant Cell* 18 3670–3685. 10.1105/tpc.106.046953 17172357 PMC1785396

[B56] Sadanandom A. Bailey M. Ewan R. Lee J. Nelis S. (2012). The ubiquitin-proteasome system: central modifier of plant signalling. *New Phytol.* 196 13–28. 10.1111/j.1469-8137.2012.04266.x 22897362

[B57] Saleh A. Withers J. Mohan R. Marques J. Gu Y. Yan S. (2015). Posttranslational modifications of the master transcriptional regulator NPR1 enable dynamic but tight control of plant immune responses. *Cell Host Microbe* 18 169–182. 10.1016/j.chom.2015.07.005 26269953 PMC4537515

[B58] Song G. Walley J. W. (2016). Dynamic protein acetylation in plant-pathogen interactions. *Front. Plant Sci.* 7:421. 10.3389/fpls.2016.00421 27066055 PMC4811901

[B59] Spange S. Wagner T. Heinzel T. Kramer O. H. (2009). Acetylation of non-histone proteins modulates cellular signalling at multiple levels. *Int. J. Biochem. Cell Biol.* 41 185–198. 10.1016/j.biocel.2008.08.027 18804549

[B60] Spoel S. H. Mou Z. Tada Y. Spivey N. W. Genschik P. Dong X. (2009). Proteasome-mediated turnover of the transcription coactivator NPR1 plays dual roles in regulating plant immunity. *Cell* 137 860–872. 10.1016/j.cell.2009.03.038 19490895 PMC2704463

[B61] Susaki E. Kaneko-Oshikawa C. Miyata K. Tabata M. Yamada T. Oike Y. (2010). Increased E4 activity in mice leads to ubiquitin-containing aggregates and degeneration of hypothalamic neurons resulting in obesity. *J. Biol. Chem.* 285 15538–15547. 10.1074/jbc.M110.105841 20190229 PMC2865332

[B62] Swaney D. L. Rodriguez-Mias R. A. Villen J. (2015). Phosphorylation of ubiquitin at Ser65 affects its polymerization, targets, and proteome-wide turnover. *EMBO Rep.* 16 1131–1144. 10.15252/embr.201540298 26142280 PMC4576982

[B63] Tacken E. J. Ireland H. S. Wang Y. Y. Putterill J. Schaffer R. J. (2012). Apple EIN3 BINDING F-box 1 inhibits the activity of three apple EIN3-like transcription factors. *AoB Plants* 2012:pls034. 10.1093/aobpla/pls034 23585922 PMC3624930

[B64] Tatham M. H. Geoffroy M. C. Shen L. Plechanovova A. Hattersley N. Jaffray E. G. (2008). RNF4 is a poly-SUMO-specific E3 ubiquitin ligase required for arsenic-induced PML degradation. *Nat. Cell Biol.* 10 538–546. 10.1038/ncb1716 18408734

[B65] Tomanov K. Zeschmann A. Hermkes R. Eifler K. Ziba I. Grieco M. (2014). Arabidopsis PIAL1 and 2 promote SUMO chain formation as E4-type SUMO ligases and are involved in stress responses and sulfur metabolism. *Plant Cell* 26 4547–4560. 10.1105/tpc.114.131300 25415977 PMC4277223

[B66] Ulrich H. D. (2005). Mutual interactions between the SUMO and ubiquitin systems: a plea of no contest. *Trends Cell Biol.* 15 525–532. 10.1016/j.tcb.2005.08.002 16125934

[B67] Van Damme P. Lasa M. Polevoda B. Gazquez C. Elosegui-Artola A. Kim D. S. (2012). N-terminal acetylome analyses and functional insights of the N-terminal acetyltransferase NatB. *Proc. Natl. Acad. Sci. U.S.A.* 109 12449–12454. 10.1073/pnas.1210303109 22814378 PMC3412031

[B68] van den Burg H. A. Takken F. L. (2010). SUMO-, MAPK-, and resistance protein-signaling converge at transcription complexes that regulate plant innate immunity. *Plant Signal. Behav.* 5 1597–1601. 21150289 10.4161/psb.5.12.13913 PMC3115111

[B69] van Wersch R. Li X. Zhang Y. (2016). Mighty dwarfs: Arabidopsis autoimmune mutants and their usages in genetic dissection of plant immunity. *Front. Plant Sci.* 7:1717. 10.3389/fpls.2016.01717 27909443 PMC5112265

[B70] Wang Y. Ha S. W. Zhang T. Kho D. H. Raz A. Xie Y. (2014). Polyubiquitylation of AMF requires cooperation between the gp78 and TRIM25 ubiquitin ligases. *Oncotarget* 5 2044–2051. 10.18632/oncotarget.1478 24810856 PMC4039143

[B71] Wang Y. S. Pi L. Y. Chen X. Chakrabarty P. K. Jiang J. De Leon A. L. (2006). Rice XA21 binding protein 3 is a ubiquitin ligase required for full Xa21-mediated disease resistance. *Plant Cell* 18 3635–3646. 10.1105/tpc.106.046730 17172358 PMC1785399

[B72] Wei Y. Diao L. X. Lu S. Wang H. T. Suo F. Dong M. Q. (2017). SUMO-targeted DNA translocase Rrp2 protects the genome from Top2-induced DNA damage. *Mol. Cell* 66 581–596. 10.1016/j.molcel.2017.04.017 .e586 28552615

[B73] Xie Y. Kerscher O. Kroetz M. B. McConchie H. F. Sung P. Hochstrasser M. (2007). The yeast Hex3.S*lx*8 heterodimer is a ubiquitin ligase stimulated by substrate sumoylation. *J. Biol. Chem.* 282 34176–34184. 10.1074/jbc.M706025200 17848550

[B74] Xu F. Huang Y. Li L. Gannon P. Linster E. Huber M. (2015). Two N-terminal acetyltransferases antagonistically regulate the stability of a nod-like receptor in Arabidopsis. *Plant Cell* 27 1547–1562. 10.1105/tpc.15.00173 25966763 PMC4456647

[B75] Xu W. H. Wang Y. S. Liu G. Z. Chen X. Tinjuangjun P. Pi L. Y. (2006). The autophosphorylated Ser686, Thr688, and Ser689 residues in the intracellular juxtamembrane domain of XA21 are implicated in stability control of rice receptor-like kinase. *Plant J.* 45 740–751. 10.1111/j.1365-313X.2005.02638.x 16460508

[B76] Yoo S. D. Cho Y. H. Tena G. Xiong Y. Sheen J. (2008). Dual control of nuclear EIN3 by bifurcate MAPK cascades in C2H4 signalling. *Nature* 451 789–795. 10.1038/nature06543 18273012 PMC3488589

[B77] Zattas D. Adle D. J. Rubenstein E. M. Hochstrasser M. (2013). N-terminal acetylation of the yeast Derlin Der1 is essential for Hrd1 ubiquitin-ligase activity toward luminal ER substrates. *Mol. Biol. Cell* 24 890–900. 10.1091/mbc.E12-11-0838 23363603 PMC3608499

[B78] Zhang Y. Goritschnig S. Dong X. Li X. (2003). A gain-of-function mutation in a plant disease resistance gene leads to constitutive activation of downstream signal transduction pathways in suppressor of npr1-1, constitutive 1. *Plant Cell* 15 2636–2646. 10.1105/tpc.015842 14576290 PMC280567

[B79] Zhou J. Lu D. Xu G. Finlayson S. A. He P. Shan L. (2015). The dominant negative ARM domain uncovers multiple functions of PUB13 in Arabidopsis immunity, flowering, and senescence. *J. Exp. Bot.* 66 3353–3366. 10.1093/jxb/erv148 25873653 PMC4449551

